# Impaired Memory B-Cell Response to Influenza Immunization in Patients With Common Variable Immunodeficiency (CVID)

**DOI:** 10.20411/pai.v6i2.405

**Published:** 2021-10-27

**Authors:** Wei Zhan, Todd Hatchette, Fengyun Yue, Jun Liu, Haihan Song, Hanqi Zhao, Stephen Betschel, Mario Ostrowski

**Affiliations:** 1 Department of Immunology, University of Toronto, Toronto, Ontario, M5S1A8, Canada; 2 Department of Pathology and Laboratory Medicine Nova Scotia Health Authority, Centre for Vaccinology, Dalhousie University, Halifax, NS B3H 1V8, Canada; 3 Keenan Research Centre for Biomedical Science of St. Michael's Hospital, Unity Health, Toronto, Ontario, M5B1W8, Canada

**Keywords:** CVID, influenza vaccine, immunodeficiency, B cells, humoral immunity, common variable immunodeficiency

## Abstract

**Background:**

Common variable immunodeficiency (CVID) is a heterogeneous primary immunodeficiency characterized by low serum antibody levels and recurrent infections. The cellular response to immunization in patients with CVID has not been fully investigated. In this study, we aimed to characterize vaccination-induced influenza-specific memory B-cell responses in CVID.

**Methods:**

Eleven individuals affected with CVID and 9 unaffected control individuals were immunized with the 2010-2011 non-adjuvanted seasonal influenza vaccine. Blood samples were collected on the day of vaccination and at week 8 and week 16 after vaccination, and PBMCs were immunophenotyped by flow cytometry. Influenza specific serology was determined using hemagglutination inhibition and microneutralization against vaccine antigens. Influenza-specific memory B-cell responses were determined by ELISpot.

**Results:**

Individuals with CVID showed wide variability in the frequency of CD19+ B cells in blood. The CVID group had significantly reduced frequencies of CD19+CD27+ memory B cells. Frequencies of circulating T follicular helper (CD4+CXCR5+) cells were similar between those with CVID and healthy controls. In terms of serology, compared to healthy controls, the CVID group overall showed significantly reduced boosting to vaccine antigens by hemagglutination inhibition and microneutralization assays at 8 weeks compared to controls and failed to maintain responses by 16 weeks compared to controls, resulting in a post-vaccination geometric mean titer (GMT) ≥ 40 to strain A/H1N1 in only 27% at 8 weeks, and 22% at 12 weeks for patients with CVID vs 78% and 75%, respectively for healthy controls. In addition, there was a GMT ≥ 40 to A/H3N2 in only 9% at 8 weeks and 22% at 12 weeks for patients with CVID vs 56% and 50%, respectively for healthy controls. Healthy participants showed significant increases in flu-specific IgM-secreting memory B cells after vaccination, whereas patients with CVID showed non-signifi-cant mild increases. Before vaccination, patients with CVID had significantly lower frequencies of background level influenza-specific IgG and IgA memory B cells. Half of the patients with CVID showed an increase in influenza-specific IgG-secreting memory B cells post vaccination, whereas the other half showed none. All control participants exhibited an increase in influenza-specific IgG-secreting B cells. None of the patients with CVID developed influenza-specific IgA memory B-cell response post vaccination, compared to 5/8 in healthy controls. At week 16, the frequency of influenza-specific memory B-cell responses decayed but to non-zero baseline in healthy controls and to zero baseline in patients with CVID.

**Conclusions:**

Together, these data demonstrate that patients with CVID respond heterogeneously, but as a group poorly, to non-adjuvanted influenza vaccine, with a subgroup unable to generate influenza-specific memory B-cell responses. No patient with CVID was able to maintain memory response for prolonged periods. Together, our results suggest a defect in Ig class switching and memory B-cell maintenance in patients with CVID during a *de novo* vaccine immune response.

## INTRODUCTION

Common variable immunodeficiency (CVID) is the most common primary immunodeficiency with a prevalence of 1/50,000 to 1/30,000 [1]. By definition, patients with CVID exhibit a clinically significant deficiency in humoral immune responses, characterized by significantly reduced serum immunoglobulin (Ig)G concentrations [2]. Most patients with CVID also exhibit reduced serum IgM and/or IgA. While most clinical manifestations of CVID are related to impairment in humoral immunity, including recurrent pulmonary, ocular, or skin infections, systemic microbial infections, and gastrointestinal symptoms related to compromised immune control of gut microbial homeostasis, some patients with CVID may also experience non-infectious symptoms such as autoimmune diseases and lymphoma [3–5]. The mainstay of treatment for CVID involves passive IgG replacement coupled with clinical surveillance of potential comorbidities [6].

The causes of CVID are unclear but likely multifactorial. Mutations in several B-cell-related genes have been observed in a minority of patients with CVID, including 10% to 15% of patients with amino acid substitutions in Transmembrane activator and CAML interactor (TACI), less than 1% with deletion in Inducible T-cell co-stimulator (ICOS), and rare mutations in CD19 and B-cell activating factor receptor (BAFFR)[7, 8]. For approximately 80% of patients with CVID, no genetic causes have been identified, and it is unclear whether factors other than gene mutations are involved.

Seasonal influenza vaccination is widely administered to prevent infection by influenza virus and reduce influenza-related mortality [9]. The commonly used, unadjuvanted, subunit, protein vaccine functions by stimulating adoptive B-cell responses to produce antibodies specific mainly to the hemagglutinin (HA) on the surface of the influenza virus. In healthy individuals, mature naive B cells become activated B cells upon stimulation, which then receive T-cell help and undergo proliferation and differentiation into either antibody-secreting plasmablasts or memory B cells. CD4^+^CXCR5^+^ T follicular helper (Tfh) cells are thought to be the major provider of B-cell help [10]. This initial wave of B-cell responses usually requires 7 days [11]. If the antigen is successfully cleared, short-lived plasmablasts may differentiate into long-lived antibody-secreting plasma cells, which are thought to be the main producer of serum Ig. Classical memory B cells typically express antigen-specific B-cell receptors at high levels on the surface. Upon secondary encounter of the specific antigen or activation via Toll-like receptor (TLR) ligands, memory B cells can rapidly differentiate into antibody-secreting cells (ASCs), thus providing protective immunity. In this regard, the B-cell response and humoral antibody formation in response to seasonal influenza vaccination has not been fully investigated in patients with CVID.

To improve our understanding of B-cell immune responses in CVID, we devised a clinical protocol to track de novo vaccine-induced B-cell immune response in patients with CVID over time.

## MATERIALS AND METHODS

### Study participants

All CVID and healthy participants were recruited under a prospective observational study approved by the research ethics committee at St. Michael's Hospital, Toronto, Canada. CVID in patients was diagnosed according to the diagnostic criteria for primary immunodeficiencies representing PAGID (Pan-American Group for Immunodeficiency) and ESID (European Society for Immunodeficiencies) [12]. Written consent was obtained from all participants. All study participants received the 2010-2011 seasonal trivalent unadjuvanted influenza vaccine containing the hemagglutinin antigens (HAs) from an A/California/7/2009 (H1N1)-like virus, an A/Perth/16/2009 (H3N2)-like virus, and a B/Brisbane/60/2008-like virus as part of routine standard of care seasonal vaccination. Peripheral blood samples were obtained by venipuncture from study participants at the time of vaccination (week 0) and subsequently at week 8 (day 56-day 63) and week 16 (day 112-day 126).

### Sample preparation

Serum samples were obtained from peripheral blood via venipuncture. Peripheral blood mono-nuclear cells (PBMCs) were isolated by standard Ficoll-Hypaque gradient centrifugation and stored in 90% heat-inactivated fetal bovine serum (FBS) plus 10% DMSO at −150°C. Before use, samples were thawed at 37°C in complete culture medium supplemented with 1% DNase (Sigma).

### Flow cytometry

PBMCs were incubated with LIVE/DEAD Fixable Violet Dead Cell Stain (Invitrogen) for 30 minutes in phosphate-buffered saline (PBS) at 4°C, washed twice, and then incubated with Human TruStain FcX (Fc Receptor Blocking) solution (BioLegend) for 5 minutes at room temperature. The cells were then incubated with a master mix of anti-human CD3, CD4, CD19, CD27, and CD185 (CXCR5) fluorescent antibodies (BioLegend) in PBS + 2% FBS for 30 minutes at 4°C and washed twice before fixation in 2% formaldehyde. Samples were acquired in a FACSCanto cytometer (BD) and analyzed in FlowJo version 10.7.1. Compensation was performed using single-stained CompBead Anti-Mouse Ig, κ/Negative Control Particle Set (BD) and ArC Amine Reactive Compensation Bead kit (Invitrogen).

### Serum influenza neutralization titers

Sera were examined for influenza hemagglutination inhibition (HAI) against vaccine antigens (A/California/7/2009 (H1N1)-like HA, and A/Perth/16/2009 (H3N2)-like HA) and microneutralization (MN) to A/California/7/09 H1N1 virus at the center for vaccinology, Dalhousie University, using WHO standardized protocols as previously described [13–15]. Sera for HAI testing was first treated with receptor destroying enzyme (RDE; Denka Seiken, Tokyo, Japan) with an overnight incubation at 37°C to remove non-specific inhibitors. Results of HAI and MN are expressed as geometric mean titers (GMT) of the reciprocal of the highest dilution that hemagglutinates or neutralizes virus.

### ELISpot assay

To evaluate the memory B-cell response, PBMCs were stimulated for 4 days with Staphylococcus aureus Cowan (SAC) and CpG oligonucleotide and then used to measure frequencies of antibody-secreting cells (ASCs) by ELISpot. Acrowell polyvinylidene fluoride filter plates (Pall, Port Washington, New York, USA) were coated with the nonadjuvanted vaccine preparation. Bound antibodies were detected using alkaline phosphatase conjugated antihuman IgG and peroxidase conjugated antihuman IgA (KPL, Gaithersburg, Maryland, USA) with subsequent development using alkaline phosphatase and peroxidase substrate kits (Vector Laboratories, Burlingame, California, USA). The frequency of influenza-specific antibody-secreting B cells was evaluated as the number of influenza-specific ASCs divided by the number of total ASCs.

### Statistical analysis

The Mann-Whitney test was used to compare 2 groups without normal distribution. The Wilcox-on matched pairs test was used to compare data obtained from the same individual at different time points. All statistical analyses were done using Prism (GraphPad). *P* < 0.05 was considered significant.

## RESULTS AND DISCUSSION

### Study design and timeline

Eleven patients with CVID diagnosed as in [12] and 9 healthy individuals were vaccinated with 2010-2011 seasonal trivalent unadjuvanted subunit protein influenza vaccine containing an A/California/7/2009 (H1N1)-like virus HA, an A/Perth/16/2009 (H3N2)-like virus HA, and a B/Brisbane/60/2008-like virus HA. Peripheral blood samples were obtained from participants at the time of vaccination (week 0) to assess the background baseline influenza-specific response from previous exposures to HA antigen, and at week 8 and week 16 post-vaccination to assess the development of memory response and its maintenance, respectively. There were no significant differences in age or sex between the patients with CVID and controls (Table 1). Further baseline immune data of CVID participants are in Supplemental Table 1. Subject 7 was of female gender with very low B-cell numbers, with no other identified cause of immunodeficiency and reflects a minor subset of individuals with CVID [12].

**Table 1.** Demographic characteristics of the study participants.

	CVID	Healthy
N	11	9
Age in years, median (range)	39 (19-49)	29 (23-42)
Sex (F/M)	4/7	2/7

### Patients with CVID had reduced frequencies of circulating memory B cells

**Figure 1.**
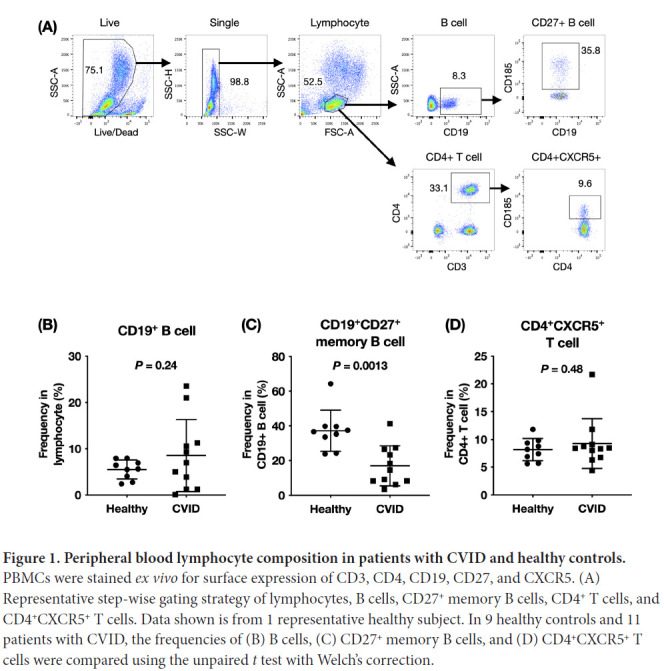
**Peripheral blood lymphocyte composition in patients with CVID and healthy controls.** PBMCs were stained *ex vivo* for surface expression of CD3, CD4, CD19, CD27, and CXCR5. (A) Representative step-wise gating strategy of lymphocytes, B cells, CD27^+^ memory B cells, CD4^+^ T cells, and CD4^+^CXCR5^+^ T cells. Data shown is from 1 representative healthy subject. In 9 healthy controls and 11 patients with CVID, the frequencies of (B) B cells, (C) CD27^+^ memory B cells, and (D) CD4^+^CXCR5^+^ T cells were compared using the unpaired *t* test with Welch's correction.

The near-normal IgM-secreting B-cell response and defective IgG-secreting and IgA-secreting B-cell responses in patients with CVID could indicate a defect in memory B-cell differentiation and antibody isotype class switching. We examined the composition of circulating B cells and T cells in patients with CVID. As shown in Figure 1, the frequencies of CD19^+^ B cells in PBMCs in healthy individuals made up between 2.4% to 8.0% of total lymphocytes. In patients with CVID, the percentage of circulating B cells in lymphocytes were highly variable (0.101% to 23.5%). When we examined the memory B cells within the B-cell compartment, we found a marked decrease in the percentages of CD19^+^CD27^+^ memory B cells in patients with CVID compared to healthy controls. Follicular helper T (Tfh) cells are thought to be the main B-cell help provider in germinal center development and circulating CD4^+^CXCR5^+^ T cells share functional attributes of germinal center Tfh cells [16]. The frequencies of circulating CD4^+^CXCR5^+^ T cells in total T cells were not significantly different between patients with CVID and healthy controls (Figure 1D).

### Patients with CVID have reduced serum Ig response to influenza vaccination

**Figure 2.**
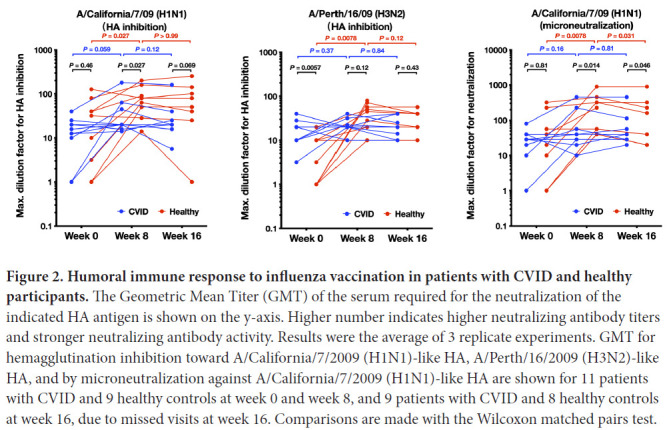
**Humoral immune response to influenza vaccination in patients with CVID and healthy participants.** The Geometric Mean Titer (GMT) of the serum required for the neutralization of the indicated HA antigen is shown on the y-axis. Higher number indicates higher neutralizing antibody titers and stronger neutralizing antibody activity. Results were the average of 3 replicate experiments. GMT for hemagglutination inhibition toward A/California/7/2009 (H1N1)-like HA, A/Perth/16/2009 (H3N2)-like HA, and by microneutralization against A/California/7/2009 (H1N1)-like HA are shown for 11 patients with CVID and 9 healthy controls at week 0 and week 8, and 9 patients with CVID and 8 healthy controls at week 16, due to missed visits at week 16. Comparisons are made with the Wilcoxon matched pairs test.

The serum Ig anti-influenza response in patients with CVID and healthy controls at the day of vaccination (week 0), and at week 8 and week 16 post-vaccination was performed. The GMT required for antigen neutralization was determined in the serum (Figure 2). A higher GMT indicates stronger neutralizing antibody activity. During the study, 12-week plasma was not obtained from 2 patients with CVID and 1 healthy control and thus, these time points are not included in the analyses.

For A/California/7/09 (H1N1)-like HA (Figure 2A), in healthy controls, the week 8 GMT was significantly higher than the week 0 GMT, while the week 16 GMT was comparable to the week 8 GMT, indicating that neutralizing antibody toward A/California/7/09 (H1N1)-like HA was induced following vaccination, and maintained to week 16. In patients with CVID, on the other hand, the week 8 GMT trended to be higher than the week 0 GMT, but did not reach statistical significance. No difference between week 8 and week 16 GMT was observed in patients with CVID. Between healthy controls and patients with CVID, the week 0 GMT was not significantly different, but the week 8 GMT titer was 2-fold higher in healthy participants vs those with CVID (mean 85 vs 40, respectively, *P*<0.05). The week 16 GMT trended to be higher in healthy controls compared to patients with CVID (mean of 87 vs 36, respectively, *P*=0.069). Thus, patients with CVID induced weaker HAI neutralization titers to A/California/7/09 H1N1 compared to controls by week 8 and 16.

For healthy controls, the GMT using HAI to A/Perth/16/2009 (H3N2) (Figure 2B), at week 8 was significantly higher than at week 0, and the week 16 GMT was comparable to the week 8 GMT. In the CVID group, we did not see significant boosting of HAI titers to A/Perth/16/09 H3N2 antigen as there were no significant differences between week 0 and week 8 GMT, or between week 8 and week 16 GMT. Interestingly, the GMT at week 0 baseline was significantly higher in patients with CVID than in healthy controls (mean 18 vs 6, *P*<0.05), which might represent previous IVIG therapy in the CVID group. However, at week 8, the GMT was 2-fold higher in the healthy vs CVID groups, although this did not achieve statistical significance using non-parametric statistics (mean 41 vs 22, respectively).

Similarly, using microneutralization assays to A/California/7/09 H1N1-like virus, we observed significant boosting of neutralization responses in healthy individuals which waned somewhat by 16 weeks, but there was no significant boosting for the CVID group (Figure 2C) (mean 8-week titer of healthy vs individuals with CVID, 269 vs 88, respectively, *P*<0.05).

**Figure 3.**
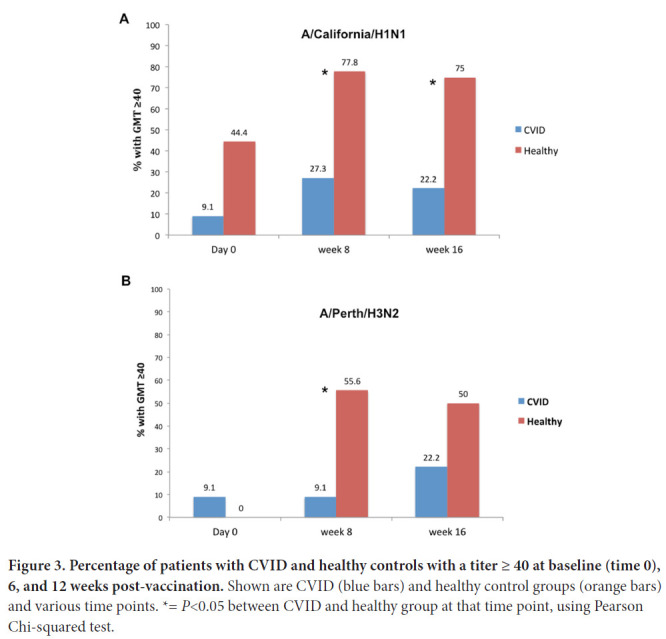
**Percentage of patients with CVID and healthy controls with a titer ≥ 40 at baseline (time 0), 6, and 12 weeks post-vaccination.** Shown are CVID (blue bars) and healthy control groups (orange bars) and various time points. *= *P*<0.05 between CVID and healthy group at that time point, using Pearson Chi-squared test.

A HAI titer of ≥ 1/40 serum dilution (GMT ≥ 40) has been considered to be protective for influenza [17]. The % of CVID vs healthy individuals to have GMT ≥ 40 for A/California/7/09 H1N1 at 6 weeks was 27% vs 78%, respectively (*P*<0.05, Pearson Chi-squared test) and at 12 weeks, was 22% vs 75%, respectively (*P*<0.05, Pearson, Chi-squared test) (Figure 3A); for A/Perth/16/2009 (H3N2) the % of individuals with CVID vs healthy individuals to have GMT ≥ 40 at 6 weeks was 9% vs 56%, respectively, (*P*<0.05, Pearson, Chi-squared test) and at 12 weeks, was 22% vs 50% respectively (*P*=n.s.) (Figure 3B).

Overall, these results indicate that neutralizing antibody in healthy participants typically increased following vaccination, while a very blunted effect was seen in the CVID group as a whole.

### Patients with CVID have impaired memory B-cell response after vaccination

Since all patients with CVID received IgG replacement therapy during the study, it is possible that neutralizing Ig detected in the serum was from IgG in the IVIG replacement. To resolve this issue, we examined the Ig response from stimulated B cells collected from healthy controls and patients with CVID. We used an ELISpot assay where B cells isolated from PBMCs were stimulated using SAC and CpG, and the frequency of influenza-specific B cells in total ASCs was measured.

**Figure 4.**
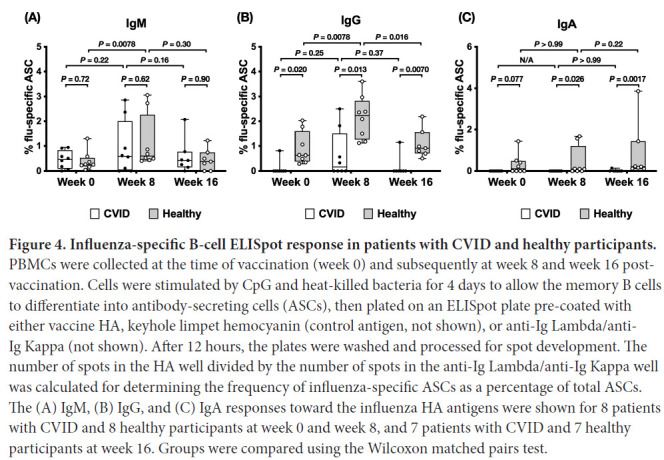
**Influenza-specific B-cell ELISpot response in patients with CVID and healthy participants.** PBMCs were collected at the time of vaccination (week 0) and subsequently at week 8 and week 16 post-vaccination. Cells were stimulated by CpG and heat-killed bacteria for 4 days to allow the memory B cells to differentiate into antibody-secreting cells (ASCs), then plated on an ELISpot plate pre-coated with either vaccine HA, keyhole limpet hemocyanin (control antigen, not shown), or anti-Ig Lambda/anti-Ig Kappa (not shown). After 12 hours, the plates were washed and processed for spot development. The number of spots in the HA well divided by the number of spots in the anti-Ig Lambda/anti-Ig Kappa well was calculated for determining the frequency of influenza-specific ASCs as a percentage of total ASCs. The (A) IgM, (B) IgG, and (C) IgA responses toward the influenza HA antigens were shown for 8 patients with CVID and 8 healthy participants at week 0 and week 8, and 7 patients with CVID and 7 healthy participants at week 16. Groups were compared using the Wilcoxon matched pairs test.

The frequencies of IgM-secreting influenza-specific B cells were comparable between patients with CVID and healthy participants at baseline (Figure 4A); however, in patients with CVID, no significant differences between week 0 and week 8, and between week 8 and week 16 were found. In healthy participants, the week 8 frequency of IgM-secreting influenza-specific B cells was significantly higher than the week 0 frequency of IgM-secreting influenza-specific B cells, while no significant difference between week 8 and week 16 was observed. Thus, healthy controls tended to boost their IgM-secreting influenza-specific B-cell response to a greater extent than the CVID group. The frequencies of IgG-secreting influenza-specific B cells (Figure 4B), on the other hand, were significantly lower in patients with CVID than in healthy individuals throughout the study. Only 1 patient with CVID at week 0, 4 (4/8), or half of the patients with CVID at week 8, and 1 patient with CVID at week 16 presented IgG-secreting influenza-specific B cells, while all healthy controls presented IgG-secreting influenza-specific B cells. In patients with CVID, no significant differences between week 0 and week 8, and between week 8 and week 16 were found. In healthy participants, the week 8 frequency of IgG-secreting influenza-specific B cells was significantly higher than the week 0 and the week 16 frequencies of IgG-secreting influenza-specific B cells. In patients with CVID, no IgA-secreting influenza-specific B cells could be found, except in 1 sample at week 16 (Figure 4C). In healthy controls, 4 out of 8 at week 0, 5 out of 8 at week 8, and 7 out of 7 at week 16 presented detectable IgA-secreting influenza-specific B cells. No significant differences between week 0 and week 8, and between week 8 and week 16, could be found. Thus, when examining memory B-cell populations, the CVID group had almost undetectable IgG and IgA secreting influenza-specific memory cells pre-immunization and had severely blunted responses post vaccination with a return to almost undetectable levels by 16 weeks after vaccination. Of note is that PBMCs for ELISpot for 3 patients with CVID and 1 healthy control were not available for weeks 0, 8, and 12. As well, 12-week PBMCs were not available for 1 patient with CVID and 1 healthy control.

For all individuals enrolled (combining CVID and healthy), we saw positive correlations between 8-week influenza HA antigen specific IgM and IgG B cell ELISpots and 8 week HAI and micro-neutralization titers (Supplemental Figure 1).

In this study, we have shown that HAI and MN antibody titers in healthy individuals typically increase following vaccination, whereas patients with CVID as a group had significantly blunted responses or no responses. Our findings are similar to those of van Assen et al [18], in which all 18 of their patients with CVID failed to develop increased GMT titers to A/H1N1, A/H3N2, or B influenza strains post trivalent vaccination. Unexpectedly, we found that for HAI using / Perth/16/2009 (H3N2)-like virus, patients with CVID had a higher GMT at week 0 baseline compared to healthy controls. Of note, the annual influenza vaccination is free and encouraged in Canada [19], the country of this study. Although some individuals might present high GMT at baseline (week 0) due to previous vaccination or infection with similar strains, this would be unlikely for patients with CVID. However, the higher baseline A/H3N2 titer in the CVID group could be explained by the fact that patients with CVID received passive IVIG during the study, which could have contained influenza-specific IgG; however, van Assen et al found no effect of passive Ig on influenza titers in participants with CVID in their cohort [18]. Patients with CVID are followed more closely in the clinic than members of the general population and thus may be immunized against influenza more frequently. Alternatively, these individuals may be at risk of more frequent influenza infections thus elevating baseline influenza antibody levels. Further studies should be undertaken to determine how frequently patients with CVID acquire asymptomatic and symptomatic influenza infection compared to the general population, and how their immune responses are affected. Of note, we observed the greatest differences in microneutralization titers between the 2 groups compared to HAI titers. The microneutralization assay has been shown to be more sensitive for seroprotection than HAI [20], and this may reflect greater avidity of antibodies in the control group, which would require further study.

In this study, we demonstrated several memory B-cell response defects in patients with CVID. First, while the influenza-specific IgM memory B-cell response in patients with CVID is near normal, with 6 out of 7 patients with CVID responding at week 8 with only 1 patient having no influenza-specific IgM response, the concurrent IgG response is severely defective, with only 3 out of 8 responding at week 8 and a fourth by week 12, and 4 out of 8 had no influenza-specific IgG memory B cells. This indicated that while most patients with CVID were able to generate antigen-specific memory B-cell responses, a defect in class-switching mechanisms in patients with CVID prevented the generation of IgG-expressing memory B cells. Second, although the frequency of influenza-specific IgG-secreting B cells was downregulated at week 16 in healthy controls, all healthy controls still maintained detectable levels of influenza-specific IgG-secreting cells at week 16. This suggests that memory B cells may be recruited to become long-lived plasma cells thus, producing prolonged production of antibody. In patients with CVID, the 3 individuals who had upregulated frequencies of influenza-specific IgG-secreting cells by week 8 could not maintain the response at week 16, indicating a defect in the maintenance of antigen-specific memory B cells in CVID. This is reflected by the lower frequencies of influenza-specific memory responses at week 8. In addition, although, we did not investigate it in this study, the lack of prolonged IgG levels at week 16 could suggest defects in plasma-cell differentiation of the memory cells. Further work in this regard is needed. Third, no influenza-specific IgA-secreting memory B cells were observed in patients with CVID except for 1 patient at week 16. It is interesting that subunit influenza vaccine induced circulating IgA ASC in our healthy cohort, since it is given intramuscularly, however, others have also shown subunit intramuscular vaccines can induce comparable IgA responses in blood to live attenuated vaccines [21]. The reasons for this are unclear, but it has previously been suggested that prior influenza infections may have primed the IgA response [21]. To summarize, these data suggest defects in antibody isotype class switching and in memory B-cell maintenance in patients with CVID, which will require future study. The differences in response patterns between individuals within the CVID group indicate that the disease is caused by multiple factors.

We attempted to investigate the potential underlying causes for observed defects by examining the circulating lymphocyte composition by flow cytometry. The most striking pattern is the lower frequency of circulating CD19^+^CD27^+^ memory B cells in patients with CVID. Also, the percentages of total CD19^+^ B cells in patients with CVID were highly variable. The differentiation of memory B cells requires coordinated interactions between antigen-presenting cells, B cells, and Tfh cells. Tfh cells in lymph nodes are characterized by the expression of transcription factor Bcl-6 and surface expression of CXCR5 and are needed for providing survival signals to B cells during germinal center differentiation. Since we observed defects in class-switching and memory B-cell maintenance, we hypothesized that altered Tfh function and regulation might be implicated in CVID pathogenesis. Circulating CD4^+^CXCR5^+^ T cells were found to have similar roles and are thought to be the peripheral counterpart of lymph node Tfh cells. In this study, we did not find a difference in the frequencies of circulating CD4^+^CXCR5^+^ T cells between patients with CVID and healthy controls, except for 1 patient with CVID with very high CD4^+^CXCR5^+^ T-cell frequency. Whether Tfh cells are functionally different in CVID lymph nodes is currently unknown.

Our current studies suggested that unadjuvanted seasonal influenza vaccination is unable to provide long-lasting protection against influenza for patients with CVID. It has been suggested that females have been shown to respond more robustly to influenza vaccines [22]. However, this is unlikely to have caused the differences we observed since the CVID group had a higher female-to-male ratio. Since IVIG can also be immunoregulatory there is the possibility that IVIG may affect immunization. In this regard, there is no known documented interaction between IVIG and inactivated vaccines [23]. Whether administration of adjuvanted vaccines can help to stimulate memory B-cell responses in CVID patients is currently unknown but should be evaluated.

## SUPPLEMENTARY DATA

Supplementary materials are available at the Pathogens and Immunity website. Supplementary data may be provided by the authors to benefit the reader. Supplementary data are not copyedited and are the sole responsibility of the authors. Questions or comments related to supplementary materials should be addressed to the corresponding author
