# Supplementary material for: Impaired Memory B-Cell Response to Influenza Immunization in Patients With Common Variable Immunodeficiency (CVID)
Source: Pathog Immun. 2021 Oct 27;6(2):105–18. doi: 10.20411/pai.v6i2.405 (PMC8714177; doi:10.20411/pai.v6i2.405)
Supplement: Supplemental Table 1 [file pai-6-105-s01.pdf]

PATHOGENS AND IMMUNITY
IN ASSOCIATION WITH CASE WESTERN RESERVE UNIVERSITY AND THE RICHARD J. FASENMYER FOUNDATION

**Supplemental Table 1: Baseline characteristics of CVID participants**

| CVID study participant # | Baseline Serum IgG (7.51-15.6) g/L | Baseline Serum IgA (0.82-4.53) g/L | Baseline Serum IgM (0.46-3.04) g/L | Baseline CD19 cells (0.135-0.447) 10^9/L | Ab Response to Pneumovax? |
|---|---|---|---|---|---|
| 1 | 1.96 | <0.1 | <0.2 | 0.249 | none |
| 2 | 3.81 | 0.8 | 2.73 | 0.039 | n/a |
| 3 | 2.23 | <0.1 | 0.25 | 0.265 | n/a |
| 4 | n/a | <0.2 | <0.21 | 0.565 | n/a |
| 5 | <0.33 | <0.07 | <0.04 | 0.061 | n/a |
| 6 | 4.08 | <.3 | 1.12 | 0.315 | none |
| 7 | n/a | <0.2 | <0.21 | 0.001 | n/a |
| 8 | n/a | <0.1 | <0.2 | 0.041 | n/a |
| 9 | <4.0 | <0.21 | <0.2 | 0.115 | n/a |
| 10 | <0.33 | <0.07 | <0.09 | 0.124 | none |
| 11 | 0.91 | <0.07 | 0.61 | 0.173 | n/a |

n/a= not available

Zhan W, Hatchette T, Yue F, Liu J, Song H, Zhao H, Betschel S, Ostrowski M. Impaired Memory B-Cell Response to Influenza Immunization in Patients With Common Variable Immunodeficiency (CVID). *Pathogens and Immunity*. 2021;6(2): 105–118. doi: 10.20411/pai.v6i2.405
