# Supplementary material for: Impaired Memory B-Cell Response to Influenza Immunization in Patients With Common Variable Immunodeficiency (CVID)
Source: Pathog Immun. 2021 Oct 27;6(2):105–18. doi: 10.20411/pai.v6i2.405 (PMC8714177; doi:10.20411/pai.v6i2.405)
Supplement: Supplemental Figure 1 [file pai-6-105-s02.pdf]

PATHOGENS AND IMMUNITY
IN ASSOCIATION WITH CASE WESTERN RESERVE UNIVERSITY AND THE RICHARD J. FASENMYER FOUNDATION

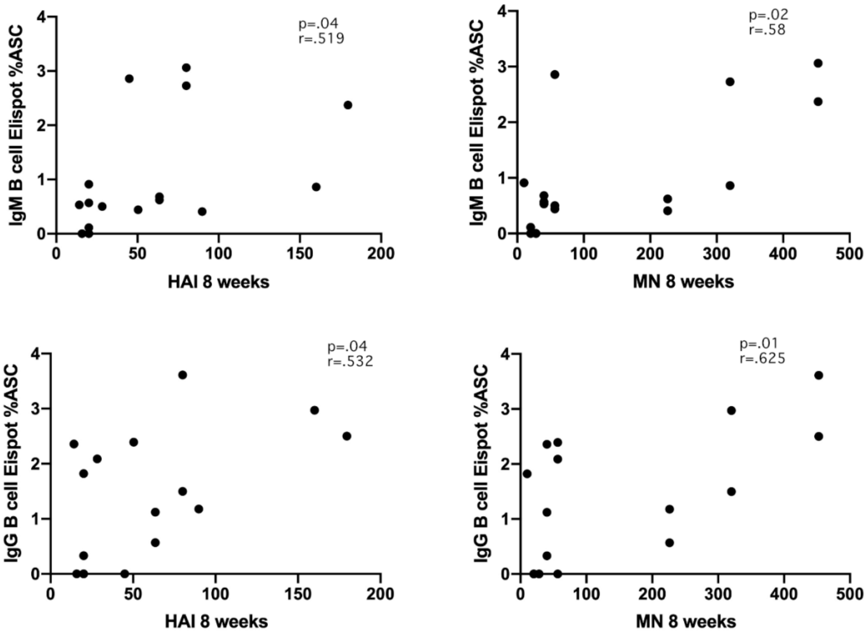

Supplemental Figure 1: Shown are correlations between 8 week IgM and IgG influenza HA antigen specific B cell ELISpot with 8 week serum HAI and microneutralization (MN) titers for all participants (CVID and healthy) tested. Analysis used spearman rank correlation.

Zhan W, Hatchette T, Yue F, Liu J, Song H, Zhao H, Betschel S, Ostrowski M. Impaired Memory B-Cell Response to Influenza Immunization in Patients With Common Variable Immunodeficiency (CVID). *Pathogens and Immunity*. 2021;6(2): 105–118. doi: 10.20411/pai.v6i2.405
